# A Plant DJ-1 Homolog Is Essential for *Arabidopsis thaliana* Chloroplast Development

**DOI:** 10.1371/journal.pone.0023731

**Published:** 2011-08-23

**Authors:** Jiusheng Lin, Tara J. Nazarenus, Jeanine L. Frey, Xinwen Liang, Mark A. Wilson, Julie M. Stone

**Affiliations:** 1 Department of Biochemistry, University of Nebraska, Lincoln, Nebraska, United States of America; 2 Redox Biology Center, University of Nebraska, Lincoln, Nebraska, United States of America; 3 Center for Plant Science Innovation, University of Nebraska, Lincoln, Nebraska, United States of America; Ecole Normale Superieure, France

## Abstract

Protein superfamilies can exhibit considerable diversification of function among their members in various organisms. The DJ-1 superfamily is composed of proteins that are principally involved in stress response and are widely distributed in all kingdoms of life. The model flowering plant *Arabidopsis thaliana* contains three close homologs of animal DJ-1, all of which are tandem duplications of the DJ-1 domain. Consequently, the plant DJ-1 homologs are likely pseudo-dimeric proteins composed of a single polypeptide chain. We report that one *A. thaliana* DJ-1 homolog (AtDJ1C) is the first DJ-1 homolog in any organism that is required for viability. Homozygous disruption of the *AtDJ1C* gene results in non-viable, albino seedlings that can be complemented by expression of wild-type or epitope-tagged *AtDJ1C*. The plastids from these *dj1c* plants lack thylakoid membranes and granal stacks, indicating that AtDJ1C is required for proper chloroplast development. *AtDJ1C* is expressed early in leaf development when chloroplasts mature, but is downregulated in older tissue, consistent with a proposed role in plastid development. In addition to its plant-specific function, AtDJ1C is an atypical member of the DJ-1 superfamily that lacks a conserved cysteine residue that is required for the functions of most other superfamily members. The essential role for *AtDJ1C* in chloroplast maturation expands the known functional diversity of the DJ-1 superfamily and provides the first evidence of a role for specialized DJ-1-like proteins in eukaryotic development.

## Introduction

The DJ-1 superfamily comprises a diverse set of proteins that are present in most organisms. Despite this broad distribution, the overwhelming majority of studies on DJ-1 superfamily proteins have focused on the human protein and its closely related animal homologs due to the involvement of human DJ-1 in parkinsonism and various cancers [1], [2], [3]. A variety of possible biochemical functions for human DJ-1 have been suggested, including RNA binding, transcriptional regulation, involvement in the Akt/PTEN/PI3K, Nrf2, and ASK1 signaling pathways, inhibition of cytotoxic protein aggregation, and glutathione metabolism [3], [4], [5], [6], [7], [8], [9], [10], [11], [12], [13]. DJ-1 has an established role in the cellular defense against oxidative stress, and the mitochondrion appears to be an important site of DJ-1 action [14], [15], [16], [17]. At the molecular level, the cytoprotective function of human DJ-1 has been shown to require both homodimerization and the presence of a conserved cysteine residue that is oxidized under stress conditions [14], [18], [19], [20]. Other members of the DJ-1 superfamily have also been characterized, and although several appear to be involved in the cellular response to stress, none has been as thoroughly studied as the human protein. In prokaryotes, there is strong evidence that more distant DJ-1 homologs have diverse functions, such as DJ-1 superfamily enzymes with isocyanide hydratase activity in the pseudomonads [21], [22], protease activity in certain archaea [23], or DNA binding proteins containing a DJ-1 domain fused to a helix-turn-helix motif in many bacteria [24], [25]. However, in eukaryotes, it remains unclear if what has been learned about animal DJ-1 pertains more generally to other eukaryotic DJ-1 homologs, particularly in the plant kingdom.

Plants contain several DJ-1 superfamily proteins, only two of which have been characterized to date. These proteins, from *Arabidopsis thaliana* and *Brassica rapa*, are both implicated in oxidative stress response, suggesting that plant and animal DJ-1 proteins may have related cellular functions [26], [27]. Plants offer unique advantages for comparative study of eukaryotic protein function, because they share many features in common with animal systems, while exhibiting major differences as a result of diverging from the animal lineage approximately 1.5 billion years ago. Like animals, plants contain mitochondria and are subject to oxidative stress, two important aspects of human DJ-1 function. Unlike animals, however, the primary source of reactive oxygen species in plants under normal conditions is photo-oxidative stress in the chloroplast [28]. Furthermore, gene duplication is common in plants, providing an opportunity to study protein functional divergence within a single organism. *Arabidopsis thaliana* is the model system of choice for *in vivo* characterization of protein function in flowering plants [29] and has a total of three close DJ-1 homologs, named AtDJ1A-C. While AtDJ1A has been previously characterized as an oxidative stress response protein, little is known about the possible functions of the other two AtDJ1 proteins.

In this study, we show that AtDJ1C is an atypical member of the DJ-1 superfamily that lacks the conserved cysteine residue that has been shown to be critical for the function of animal DJ-1. Disruption of the *dj1c* gene (At4g34020) by T-DNA insertion in *Arabidopsis thaliana* results in albino seedlings with malformed chloroplasts, and is the first known DJ-1 sueprfamily protein that is required for viability in any organism. The essential role of DJ1C in plastid development is distinct from any currently proposed model for DJ-1 protein function and suggests that eukaryotic DJ-1 homologs are more functionally diverse than currently appreciated. The physiological importance of AtDJ1C also demonstrates that the conserved cysteine residue that is a hallmark of the DJ-1 superfamily is not essential for the functions of all superfamily members.

## Results

### Plant DJ-1 superfamily genes encode tandem duplications of DJ-1-like domains

**Figure 1:**
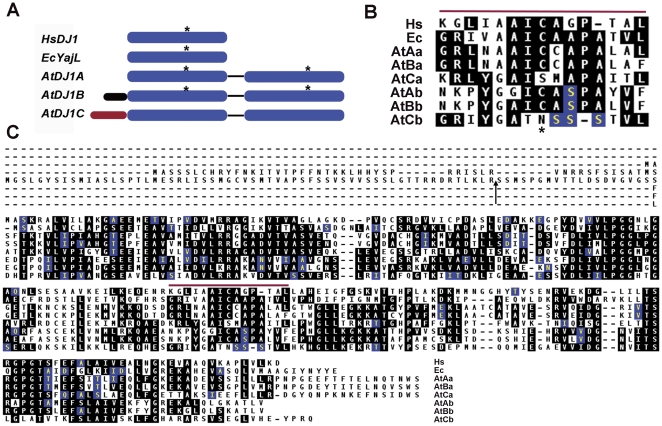
DJ-1 homologs are widespread, and plant genes encode tandem duplications of DJ-1 domains. (A) Proteins encoded by plant, animal and bacterial DJ-1 homologs, *Homo sapiens* (Hs), *Escherichia coli* (Ec) and *Arabidopsis thaliana* (At), are represented schematically. *H. sapiens* (HsDJ1), *E. coli* (EcYajL) and *A. thaliana* (AtDJ1A, AtDJ1B and AtDJ1C), DJ-1/ThiJ domains (blue) and N-terminal extensions (red or black) are represented. Calculations using various web-based programs to predict subcellular localization indicate that HsDJ-1, EcYajL and AtDJ1A have no detectable N-terminal targeting sequences, AtDJ1B targeting is ambiguous (mitochondria and/or chloroplast, black), and AtDJ1C has a chloroplast transit peptide (cTP, red). The relative position of the highly conserved cysteine modified by oxidation in HsDJ-1 (C106) is indicated with an asterisk (*). (B, C) Amino acid alignments (Clustal) of plant, animal and bacterial DJ-1 homologs were performed with DNAStar Lasergene software. N-terminal and C-terminal DJ-1 domains of the plant genes are designated a and b, respectively. A region surrounding the highly conserved cysteine (*) is shown (B), and the corresponding region in the full alignment is also indicated by a red bar (C). The N-terminal extensions found in the plant AtDJ1B and AtDJ1C proteins and predicted cleavage site for the AtDJ1C cTP (arrowhead) are also indicated (C).

The large DJ-1 superfamily is represented in nearly all organisms [24], [30], [31]. Superfamily members are predominantly either single domain proteins or fusions of a DJ-1-like domain with a helix-turn-helix motif, which is common in prokaryotes [24], [30]. In the *A. thaliana* genome, three genes encoding close homologs of human DJ-1 were identified (At3g14990, At1g53280 & At4g34020). Other, more distant DJ-1 superfamily members are also present in *Arabidopsis*, such as At3g02720, At3g54600, and At2g38860, which belong to the YhbO/PfpI clade of the DJ-1 superfamily. Interestingly, all of these plant homologs encode two tandem DJ-1-like domains, a gene architecture which appears to be unique to plant members of the superfamily [24], [30], [31]. The highly conserved cysteine residue that contributes to the redox regulation of animal DJ-1 [8], [14], [15], [18], [32] is conserved in both tandem DJ-1 domain repeats of AtDJ1A and AtDJ1B, but absent in both DJ-1 domains of AtDJ1C (Fig. 1). Therefore, AtDJ1C is an atypical member of the DJ-1 superfamily that lacks this highly conserved residue and functionally essential residue in the animal DJ-1s. In addition, both AtDJ1B and AtDJ1C have N-terminal extensions that are predicted to target the proteins to mitochondria and/or chloroplasts (Fig. 1), consistent with previous subcellular localization studies of these proteins [33].

### AtDJ1C is a chloroplast-targeted protein that is expressed in young expanding leaves with a gradient of expression

**Figure 2:**
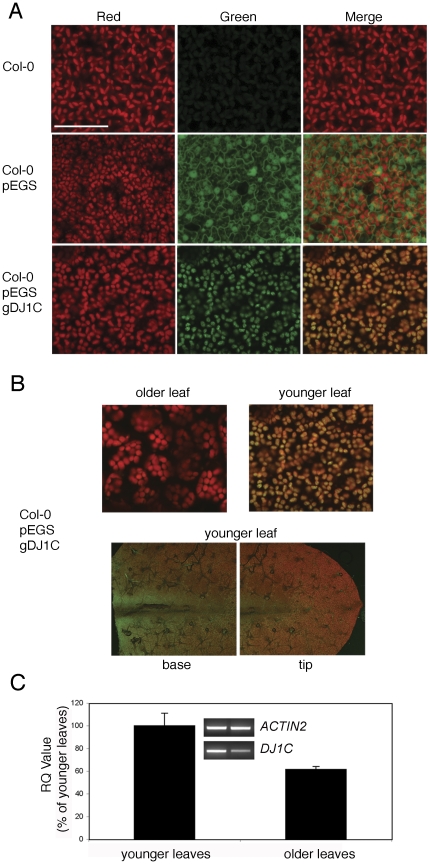
*AtDJ1C* encodes a chloroplast-targeted protein, expressed in young expanding leaves, with a gradient of expression. (A) *AtDJ1C* was cloned into the binary vector pEGS to produce an in-frame fusion to green fluorescent protein (GFP) at the C-terminus of the protein driven by the native promoter (p*DJ1C*:*gDJ1C:GFP*). Subcellular localization of DJ1C::GFP in *Arabidopsis thaliana* transgenic plants, generated by *Agrobacterium tumefaciens*-mediated transformation, was visualized by confocal microscopy. Controls included non-transgenic, wild-type Col-0 plants as negative and transgenic Col-0 plants harboring the pEGS vector (p35S:GFP) as positive controls (GFP expression is observed in both the cytoplasm and nucleus). Transgenic plants harboring the p*DJ1C*:*gDJ1C:GFP* construct show AtDJ1C-GFP expression exclusively in chloroplasts. Images were taken at 60X magnification at negative control levels. Red channel, green channel and merged images from Z-series of leaf epidermal and mesophyll cells are shown, and white bar represents 50 µm. (B) Greater AtDJ1C-GFP expression was observed in young, expanding leaves than mature, fully expanded leaves. Moreover, a gradient of expression is observed in young expanding leaves, with strongest expression near the major vein at the basal portion and weakest expression at the apical tip. Top panels show merged images at 60X magnification. Bottom panels show merged images of a young expanding leaf at low magnification. (C) Relative-quantitative RT-PCR validates live cell imaging. Total RNA isolated from leaves was reverse transcribed with oligo-dT and used as template for PCR with *ACTIN2*- and *AtDJ1C*-specific oligonucleotide primers. Relative steady-state *AtDJ1C* gene expression was determined as the ratio of *DJ1C* to *ACTIN2* (inset). Older leaves have significantly lower (∼60% p<0.05) *AtDJ1C* transcript levels than young, expanding leaves. Error bars represent SEM (n = 4).

Chloroplast transit peptides (cTPs) are sequence motifs in the N-terminal residues of proteins that direct proteins to this organelle [34], [35], [36]. *AtDJ1C* encodes a 465 amino acid protein of 51 kDa molecular weight and contains a predicted cTP at its N-terminus. Therefore, AtDJ1C is predicted to be targeted to the chloroplast (Fig. 1). To test this, transgenic plants harboring a construct producing GFP-tagged DJ1C driven by its native promoter (p*DJ1C*:*gDJ1C:GFP*) were generated and analyzed by live cell imaging and confocal microscopy. The use of the native promoter avoids potential artifacts resulting from non-physiological levels of protein expression and also permits the study of developmental regulation of AtDJ1C expression. Microscopy reveals that AtDJ1C-GFP is targeted to chloroplasts and that greater expression is observed in young, expanding leaves than mature, fully expanded leaves. This observation is confirmed by relative quantitative RT-PCR of the *AtDJ1C* transcript; older leaves have significantly lower (∼60%, p<0.05) *AtDJ1C* transcript levels than young, expanding leaves (Fig. 2). Moreover, a gradient of expression is observed in young expanding leaves, with strongest expression near the major vein at the basal portion and weakest expression at the apical tip (Fig. 2). This pattern of expression mirrors the leaf developmental gradient [37] and is correlated with the gradient of chloroplast maturation in a new leaf. In particular, etioplasts are transitioning to chloroplasts at the base of the leaf, where AtDJ1C expression is greatest, while protein levels diminish in regions of the leaf where chloroplasts have fully matured. In light of the localization of AtDJ1C to the chloroplast (see above), this suggests that the protein plays a role in chloroplast development and maturation.

### Knockout *dj1c* T-DNA insertion mutants are seedling lethal with compromised chloroplast ultrastructure

**Figure 3:**
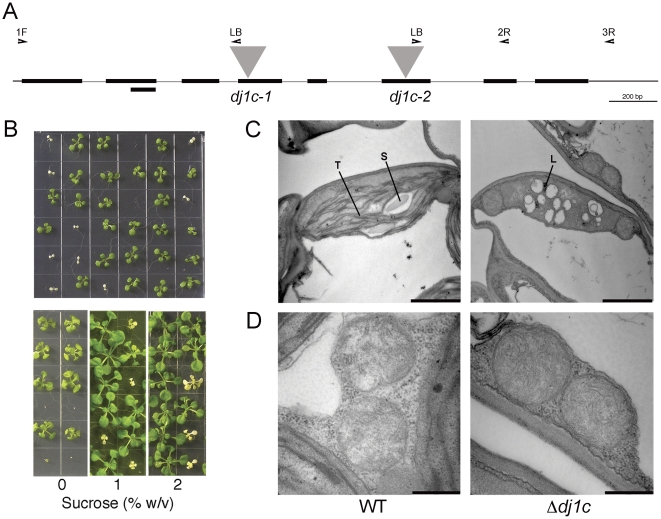
*DJ1C* is an essential gene; *dj1c* T–DNA insertions are seedling lethal with compromised chloroplast ultrastructure. (A) Schematic representation of *AtDJ1C* genomic DNA. Eight exons are represented by black bars and exon 2 alternative splicing. The T-DNA insertions in two *dj1c* null mutant alleles (*dj1c-1*, SALK_125439; *dj1c-2*, SALK_022772) are represented by gray triangles. Annealing sites for oligonucleotide primers used for PCR-based genotyping and RT-PCR are indicated (see Table S1). (B) Progeny from a plant hemizygous for the *dj1c-1* T-DNA insertion allele, indicate that one quarter of the germinated seeds are small, albino seedlings that subsequently die, as expected for single, recessive mutations. Supplementation with sucrose results in moderate growth. (C-D) Chloroplast ultrastructure is compromised, but mitochondrial ultrastructure is unaffected, in *dj1c* cotyledons. For transmission electron microscopy (TEM), thin sections of cotyledon tissue were observed under a transmission electron microscope (Hitachi H7500-I). Wild-type (WT) cotyledons show typical chloroplast structure with thylakoid membranes and granal stacks (T) and starch granules (S), whereas *dj1c* mutant (*dj1c*) cotyledons lack organized thylakoid membranes and display enhanced accumulation of lipid-rich bodies (L) and internal vesicles. Differences in mitochondrial numbers and morphology were not observed. Black bars represent 2 µm (C) or 0.5 µm (D).

The native *AtDJ1C* gene was disrupted by T-DNA insertion to determine the effect of its absence on *Arabidopsis*. Two distinct *DJ1C* T-DNA insertion alleles indicate that *AtDJ1C* is essential for *Arabidopsis* viability. T-DNA insertions responsible for two *dj1c* knockout alleles (*dj1c-1*, SALK_125439; *dj1c-2*, SALK_022772) were validated by PCR-based genotyping (Fig. 3 and Fig. S1). After crossing plants that were hemizygous for the two *dj1c* T-DNA insertion alleles, a fraction of the progeny are small, albino seedlings that subsequently die, demonstrating that *AtDJ1C* is essential for viability. The albino phenotype segregates in hemizygous crosses 3:1 for viable:albino (*dj1c-1*, n = 587, *X* ^2^ = 0.128; *dj1c-2*, n = 169; *X* ^2^ = 2.148), as expected for a single, recessive mutation. Moderate growth of the *dj1c* knockout albino seedlings can be achieved by culturing on media containing varying concentrations of sucrose, which compensates for the loss of photosynthetic capacity in *dj1c* null mutants by providing an exogenous carbon source (Fig. 3B). The improved viability of the albino homozygous *dj1c* knockout seedlings demonstrates that the lethality of the *dj1c* mutation is due to loss of photosynthetically-driven carbon fixation.

The albino seedling-lethal phenotype of the *dj1c* mutants is consistent with the chloroplast localization of GFP-tagged AtDJ1C and the expression gradient of *AtDJ1C* in leaves (Fig. 2), as all of these data suggest that AtDJ1C plays a critical role in chloroplast maturation. To investigate the impact of *dj1c* null mutants on organellar ultrastructure, thin sections of wild-type Col-0 and *dj1c* mutant cotyledon tissue were analyzed by transmission electron microscopy (TEM). Relative to wild-type chloroplasts, the *dj1c* plastids are smaller, deformed, and devoid of thylakoid membrane, granal stacks and starch granules. Large internal vesicles that resemble those found in proplastids and associated lipid-rich bodies (plastoglobules) are prevalent in *dj1c* mutant plastids (Fig. 3C). These grossly malformed chloroplasts explain the albino phenotype of the *dj1c* mutant seedlings, as these plants cannot develop mature, thylakoid-containing chloroplasts that are capable of supporting photosynthesis. In contrast, mitochondria from *dj1c* cotyledon tissue appear normal in these micrographs (Fig. 3D). These plastid ultrastructure abnormalities observed in *dj1c* mutant tissue indicate that AtDJ1C functions in early stages of differentiation of proplastids to etioplasts and chloroplasts [38], [39].

### The *dj1c* mutant seedling-lethal phenotype is rescued by an epitope-tagged version of *AtDJ1C* driven by its native promoter

**Figure 4:**
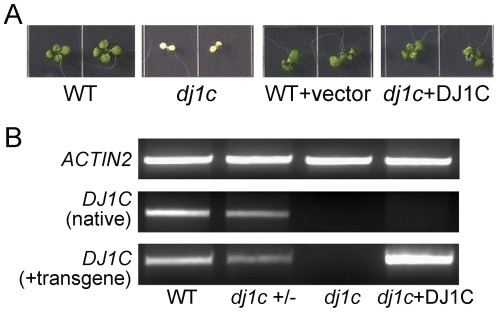
Seedling lethality of *dj1c* mutants can be complemented by epitope-tagged *AtDJ1C.* (A) Seedlings were sown on MS-agar-sucrose plates. (B) Total RNA isolated from leaves was reverse transcribed and used as template for PCR with *ACTIN2*- and *AtDJ1C*-specific oligonucleotide primers (Table S1 and Fig. 3) to determine transcript levels for the native *DJ1C* gene (3R primer anneals to the 3′UTR) and *DJ1C* plus the transgene. Genotypes were confirmed by PCR-based genotyping as in Fig. S1. Wild-type, WT; homozygous *dj1c* null mutant, *dj1c*; hemizygous *dj1c*, *dj1c* +/−; *dj1c* null mutant complemented with p*DJ1C*:*gDJ1C:GFP*, *dj1c*+DJ1C.

Our results show that two distinct, recessive *dj1c* T-DNA insertion alleles cause seedling lethality, supporting the conclusion that the phenotype is due to disruption of the *dj1c* gene. To demonstrate that albino seedling lethality is due to deficiency of the AtDJ1C protein, a complementation experiment was performed whereby transgenic plants harboring the p*DJ1C*:*gDJ1C:GFP* expression construct were crossed to the hemizygous *dj1c-1* background. The F2 progeny were all viable, and both the hemizygous and complemented *dj1c* mutants were not phenotypically distinguishable from WT (Fig. 4A). PCR-based genotyping (Fig. S1) and relative quantitative RT-PCR for native *DJ1C* and the *DJ1C* transgene (Fig. 4) verify that the seedling-lethal phenotype can be effectively complemented by a functional, GFP-tagged DJ1C. This not only establishes that absence of the AtDJ1C protein is responsible for loss of chloroplast integrity but also shows that this GFP-tagged version of AtDJ1C is functional *in vivo*.

## Discussion

Animal DJ-1 plays an important role in response to oxidative stress and mitochondrial dysfunction, and has been independently connected to parkinsonism [1] and multiple cancers [3], [40], [41]. However, despite the importance of DJ-1 for cytoprotection, the protein is not essential for viability in human, mouse, zebrafish or fly [18], [42], [43], [44], [45], [46], [47]. In contrast, our results demonstrate that at least one of the three *Arabidopsis thaliana* DJ-1 homologs (AtDJ1C) is required for proper chloroplast development and, therefore, for viability. This is the first reported example of a lethal knockout of a member of the DJ-1 superfamily and thus provides a useful system in which to investigate the function of DJ-1 in flowering plants. The requirement for AtDJ1C in chloroplast development also suggests an intriguing conserved connection between DJ-1 and energy-generating organelles in eukaryotes, as previous work has shown an important role for human DJ-1 in mitochondrial function.

Previous studies of plant DJ-1 proteins have focused on AtDJ1A and BrDJ-1, both of which are upregulated by oxidative stress. In a study of AtDJ1A, Xu et al. found that the protein conferred protection against diverse stressors and implicated both Cu-Zn superoxide dismutase and glutathione peroxidase 2 in the protective function of AtDJ1A [33]. While the biochemical details of these two plant DJ-1 proteins' functions are not known, they are similar to the animal DJ-1 homologs in conferring protection against oxidative stress. In contrast, AtDJ1C is functionally distinct from other characterized DJ-1 homologs, including its two other close paralogs in *Arabidopsis*. AtDJ1C is targeted to developing chloroplasts (Fig. 2), consistent with another report that it is found in plastids [33]. Moreover, at the sequence level, AtDJ1C is unusual because it lacks the highly conserved, oxidation-sensitive cysteine residue that was previously identified as functionally essential in several other DJ-1 homologs [14], [15], [18], [48]. The absence of this otherwise conserved cysteine in AtDJ1C indicates that oxidative regulation of DJ-1 function is not a universally conserved feature of the DJ-1 family. The absence of this cysteine residue may be related to the chloroplast localization of AtDJ1C, as cysteine content is low is chloroplast proteomes compared to the remainder of the plant cell proteome [49], [50]. The scarcity of cysteine in chloroplast-resident proteins (other than those with known redox-sensitive regulatory cysteines) may reflect an adaptation to the highly reductive redox environment of the chloroplast, which can generate large amounts of ROS that would be damaging to sulfur-rich proteins [51].

A key structural feature of all known members of the DJ-1 superfamily is that they form dimers (and sometimes higher oligomers [52]) with differing interfaces and this oligomerization is important for function in the characterized members of the superfamily [31], [53]. The plant DJ-1 proteins have a unique gene structure consisting of two DJ-1 domains in a tandem array (Fig. 1), distinguishing them from other members of the DJ-1 superfamily. The unique gene structure of the plant DJ-1-like proteins suggests that these plant proteins likely form a DJ-1-like pseudo-dimer within a single polypeptide. This interesting feature is also shared by plant genes encoding related PfPI domains as well as other plant proteins with reactive cysteines [54]. Therefore, domain duplication in plant genes encoding homologs of homodimeric proteins is common, although its evolutionary significance has not yet been fully explored. We propose that tandem domain fusions in plants, which can be readily identified from genomic data, could be used to identify probable homodimeric protein homologs in other organisms.

The results presented here show that the chloroplast-resident AtDJ1C protein plays a critical role in plant development. Although there is substantial functional divergence between the plant and animal DJ-1 homologs, the clear homology between these proteins and the wide distribution of DJ-1 proteins in other organisms indicates that they likely share some functional similarities. The transient expression of AtDJ1C in newly developing leaf tissue, its relatively low abundance, and the absence of the conserved cysteine residue do not support a likely role for AtDJ1C in ROS scavenging, protein chaperoning, or an occult enzymatic activity, three possible functions that have been suggested for animal DJ-1. However, the proposed roles of DJ-1 in cellular regulation at the transcriptional, translational, and protein interaction levels are consistent with the profound consequences of *AtDJ1C* deletion in plants and merit further investigation.

## Materials and Methods

### Plant material and growth conditions

*dj1c* T-DNA insertion mutants (SALK_125439 and SALK_022772, ecotype Col-0) were obtained from the Arabidopsis Biological Resource Center (Ohio State University, Columbus. OH). Seeds were imbibed in 0.8% agarose, sown on Metro Mix 360, incubated in darkness at 4°C for 4 d, and transferred to a temperature-controlled growth chamber (22°C) on a 12-hr-light/12-hr-dark cycle (∼100–150 mE ms^−1^ s^−1^). For growth under sterile conditions, seeds were surface-sterilized with 3% sodium hypochlorite, 0.02% Tween-20 for 10 min, rinsed with sterile H_2_O, incubated in darkness at 4°C for 4 d, sown on MS-0.8% phytagar plates (Research Products International Corp.) supplemented with sucrose (0 to 2% w/v), and incubated at 24°C under a 12-hr photoperiod.

### PCR-Based genotyping, cloning and plant transformation

*A. thaliana* genomic DNA was isolated from leaf tissue using either modified Dellaporta or “quick and dirty” DNA Prep for PCR protocols [55], [56]. For genotype determinations, various combinations of oligonucleotide primers (LB6313R-SALK, DJ1Cgeno-F and DJ1geno-R2; Table S1) were used to distinguish wild-type, hemizygous, and homozygous *dj1c* T-DNA insertion mutants. PCR was performed for 35 cycles (94°C 60 s, 56°C 60 s, 72°C 180 s) in 25 µL reactions containing 200 µM dNTPs, 1.5 mM MgCl_2_, 2% DMSO, 0.4 µM each oligonucleotide primer, Taq polymerase, 1X PCR buffer, and 100 ng of template DNA. Reaction products were visualized after electrophoresis on 1% agarose gels containing ethidium bromide. For both subcellular localization and genetic complementation, DJ1C (native promoter and coding sequence) was amplified from Col-0 genomic DNA with oligonucleotide primers DJ1CSacIF and DJ1CSmaIR (Table S1) and SuperTaq Plus (Ambion), cloned into pGEM-TEasy (Promega), and verified by DNA sequencing. To produce DJ1C with a C-terminal GFP tag driven by the native promoter, the p*DJ1C*:*gDJ1C:GFP* construct was generated by ligating the DJ1C EcoICRI/SmaI fragment into EcoICRI/BamHI-digested pEGS, a binary vector derived from pEGFP-N3 (Clontech) and pEGAD [57]. Col-0 plants were transformed with *Agrobacterium tumefaciens* strain GV3101 using the floral dip method [58]. Independent homozygous lines were analyzed.

### Genetic crosses and genetic complementation

Hemizygous *dj1c-1* (SALK_125439) was crossed with homozygous transgenic pEGSgDJ1C P3D1B. F1 generation seedlings were verified by selection for the glufosinate resistance marker carried by the pEGS-derived T-DNA (Finale™, 1∶100 v/v) and PCR-based genotyping using oligonucleotide primers oJS368 and oJS642 for the wild-type allele and oJS368 and oJS473 for the *dj1c* T-DNA insertion allele (Table S1). The F2 progeny from an individual hemizygous for *dj1c* and the pEGSgDJ1C transgene were again selected for glufosinate resistance and subjected to PCR-based genotyping. Plants homozygous for *dj1c* and either hemizygous or homozygous for the pEGSgDJ1C transgene were analyzed for complementation of the *dj1c* seedling-lethal phenotype.

### RNA isolation and RT-PCR analyses

Plants were grown in growth chambers as described above. From 3- to 4-week old plants (prior to bolting) tissue was collected from both younger and older leaves, still expanding or fully expanded, respectively. Total RNA was isolated using Trizol reagent according to manufacturer's instructions (Invitrogen), and RNA concentration was determined spectrophotometrically. Reverse transcription was performed for 1 h at 42°C in 20 µl reactions containing 1 µg total RNA, 500 ng Oligo-dT_18_ primers, 40 U RNAsin (Promega), 500 µM dNTPs, and 40 U M-MuLV reverse transcriptase (Fermentas). PCR was performed in 25 µl reactions containing 1X Econo Taq PCR buffer, 1.25 U Econo Taq PCR enzyme (Lucigen), 50 µM dNTPs and oligonucleotide primers specific for *DJ1C* or *ACTIN2* (Table S1) for 27 or 25 cycles (94°C 30 s, 55°C 30 s, 72°C 90 s), respectively. Transcripts corresponding to native *DJ1C* genes were distinguished using a reverse primer that anneals to the 3′UTR of the gene (oJS642, 3R, Table S1 and Fig. 3). The RT-PCR reactions were electrophoretically resolved by agarose gel electrophoresis and analyzed with a Bio-Rad Gel Doc and Quantity One Software (Bio-Rad). Students' t-tests (Microsoft Excel) were used for statistical analyses.

### Photography, Confocal and Transmission Electron Microscopy

Subcellular localization of GFP in stably transformed *A. thaliana* harboring the p*DJ1C*:*gDJ1C:GFP* construct was visualized by confocal laser scanning microscopy using an Olympus Fluoview 500 and analyzed using Olympus Fluoview software version 4.3 (Olympus). All images were taken at negative control levels or lower. The cellular ultrastructures of *dj1c* null mutant cotyledons were observed by transmission electron microscopy (TEM). Cotyledon tissues were prepared and analyzed essentially as described with some modifications [59]. Wild-type Col-0 and *dj1c* null mutant seedlings were fixed with 2.5% glutaraldehyde in 50 mM sodium cacodylate pH 7.4, under a vacuum for 3 hours, post-fixed in 1% osmium tetroxide in 50 mM sodium cacodylate pH 7.4 for 2 hours, dehydrated in a graduated ethanol series, and embedded in Epon 812 (Electron Microscopic Sciences). Thin sections (80–100 nm) were stained with uranyl acetate and lead citrate and observed under a transmission electron microscope (Hitachi H7500-I).

## Supporting Information

Figure S1 **PCR-based genotyping for** ***dj1c*** **mutant alleles.** To determine whether individual plants were wild type (WT), hemizygous for *dj1c* (*dj1c* +/−), or homozygous for *dj1c* in plants complemented with the DJ1C transgene (*dj1c* +DJ1C), DNA was subjected to PCR using oligonucleotide primers that anneal to regions of *DJ1C* or the T-DNA (Table S1 and Fig. 3).
(TIF)

Table S1 **Oligonucleotide primers.**
(DOC)
