# Supplementary figures and images for: A Plant DJ-1 Homolog Is Essential for Arabidopsis thaliana Chloroplast Development
Source: PLoS One. 2011 Aug 23;6(8):e23731. doi: 10.1371/journal.pone.0023731 (PMC3160306; doi:10.1371/journal.pone.0023731)

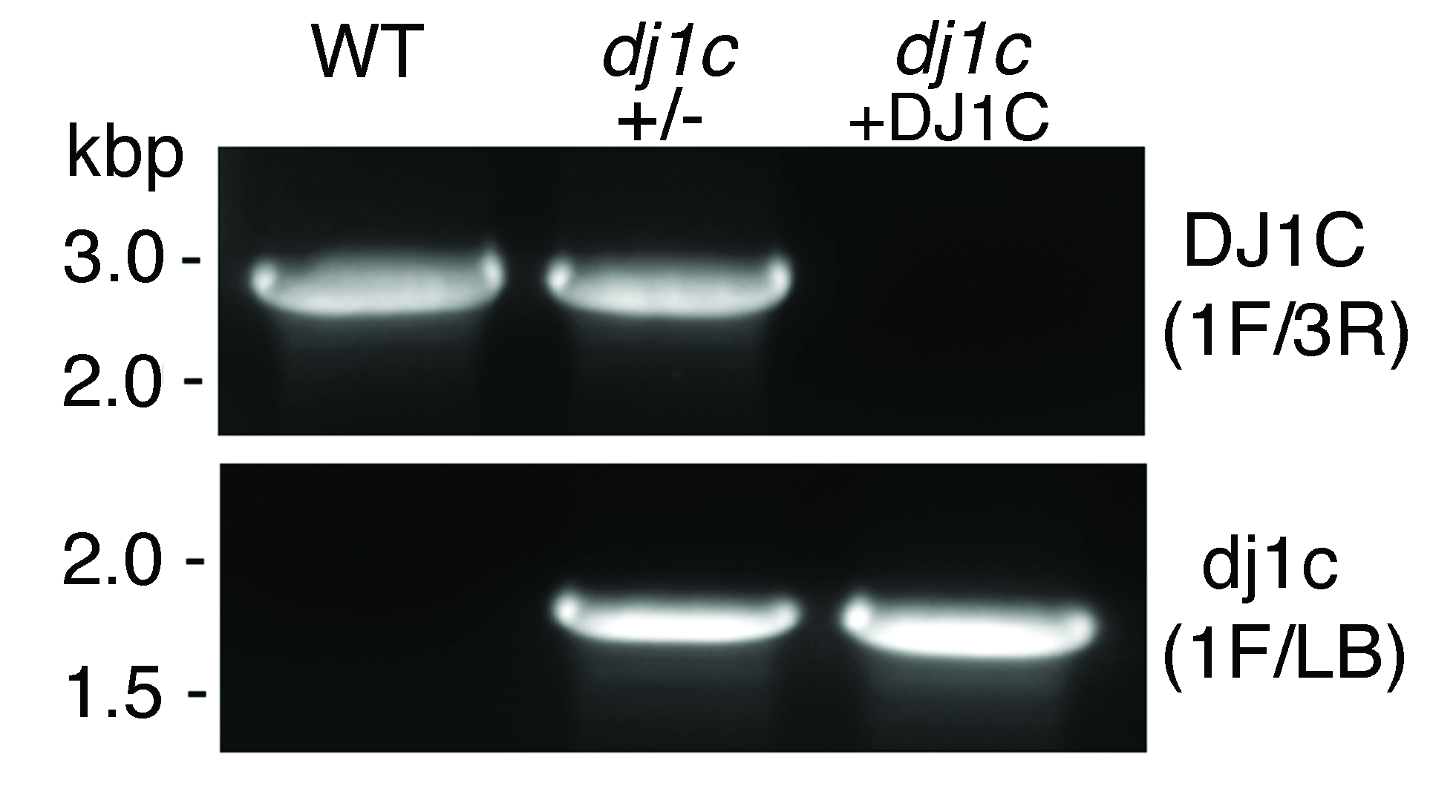

Supplement: Figure S1 — PCR-based genotyping for dj1c mutant alleles. To determine whether individual plants were wild type (WT), hemizygous for dj1c (dj1c +/−), or homozygous for dj1c in plants complemented with the DJ1C transgene (dj1c +DJ1C), DNA was subjected to PCR using oligonucleotide primers that anneal to regions of DJ1C or the T-DNA (Table S1 and Fig. 3). (TIF) [file pone.0023731.s001.tif]
