# Supplementary material for: A Plant DJ-1 Homolog Is Essential for Arabidopsis thaliana Chloroplast Development
Source: PLoS One. 2011 Aug 23;6(8):e23731. doi: 10.1371/journal.pone.0023731 (PMC3160306; doi:10.1371/journal.pone.0023731)
Supplement: Table S1 — Oligonucleotide primers. (DOC) [file pone.0023731.s002.doc]

**Table S1. Oligonucleotide primers used in this study.**

| **Designation**^1^ | **Name** | **Sequence** | **Purpose** |
| --- | --- | --- | --- |
| oJS487 | DJ1CSacIF | 5’-GAGCTCCAAAAAGGAAGAACCAGG-3' | Cloning |
| oJS488 | DJ1CSmalR | 5'-CCCGGGCACTGCCTTGGATACTCATGCAC-3' | Cloning |
| oJS368 (1F) | DJ1Cgeno-F | 5'-ATGGGGTCTTTAGGATATTCG-3' | Genotyping/RT-PCR |
| oJS482 (2R) | DJ1Cgeno2-R | 5'-CGCCCAGATTCATGTTGTTCCC-3' | Genotyping/RT-PCR |
| oJS473 (LB) | LB_6313R | 5'-TCAAACAGGATTTTCGCCTGCT-3' | Genotyping |
| oJS642 (3R) | DJ1C geno R3 | 5'-CCATCGGACCGTTATACTTCGG-3' | RT-PCR |
| oJS507 | ACT2-F | 5'-GTGCCAATCTACGAGGGTTTC-3' | RT-PCR |
| oJS508 | ACT2-R | 5'-CAATGGGACTAAAACGAAAA-3' | RT-PCR |

^1^Refers to the laboratory designation with relative annealing positions (Fig. 3) indicated in parentheses.
